# Refractive surprise after cataract surgery secondary to smeared optics of swept-source optical coherence tomography biometer: a case report

**DOI:** 10.1186/s12886-020-01629-0

**Published:** 2020-08-28

**Authors:** Francis Carr, Vinod Gangwani

**Affiliations:** Department of Ophthalmology, Ashford and St Peter’s Hospitals, Surrey, KT16 0PZ UK

**Keywords:** Biometer error, Biometry, Swept-source optical coherence tomography, Refractive surprise, Cataract surgery, Case report

## Abstract

**Background:**

Inaccurate biometry can lead to the wrong intraocular lens implantation and result in refractive surprise following cataract surgery. It is important to be sceptical of biometry results that do not match the refractive or clinical picture and ask for it to be repeated.

**Case presentation:**

We present a unique cause of refractive surprise in a patient undergoing cataract surgery. Pre-operative refraction demonstrated hypermetropia, yet swept-source optical coherence tomography (SS-OCT) biometry repeatedly calculated the axial length as > 35.00 mm in both eyes. The patient underwent phacoemulsification and intraocular lens insertion using the provided biometry calculations, however post-operatively the patient had a + 14.00 dioptre refractive surprise. Analysis of biometry performed on the same day identified other patients with exaggerated axial lengths, supporting the theory that the biometer’s smeared optical surface was responsible. Following servicing of the machine, repeat biometry of the patient calculated the axial length consistent with a hypermetrope (21.67 mm) and the intraocular lens exchange was successful in correcting the refractive error.

**Conclusions:**

Ensure the optical surfaces of the biometer are cleaned regularly, and consider repeating biometry on separate days if repeat biometry still is not in keeping with the refractive or clinical picture. Additionally, re-confirm the axial length with another modality.

## Background

Cataract surgery with intraocular lens implantation most often attempts to achieve a refractive result close to emmetropia and reduce spectacle dependence. To obtain this, an accurate intraocular lens calculation is compulsory and axial length measurement is perhaps the most influential parameter in this calculation [1]. Axial length is commonly measured using partial coherence interferometry and recently with SS-OCT, as it offers advantages over ultrasound biometry, such as greater precision, unaffected by velocity estimates and measuring along the visual axis [2–4].

Despite the evolving technologies, formulae and protocols, the intended post-operative refractive target is not always achieved [5]. This is termed a ‘refractive surprise’ and is a source of patient disappointment due to unmet expectations [6–8]. We report a case of wrong intraocular lens implantation during cataract surgery, the result of consistent, yet repeatedly incorrect, axial length measurements secondary to the biometer’s smeared optical surface.

## Case presentation

A 78-year-old hypermetropic woman was recommended cataract surgery in both eyes. She was unhappy with deteriorating quality of vision, particularly in the right eye. Her corrected distance visual acuity was 20/40 in the right eye and 20/30 in the left eye. Her manifest refraction was + 3.75 DS in the right eye and + 4.00 DS / + 0.50 DC × 79 in the left eye. Anterior segment examination demonstrated bilateral cataract (nuclear sclerotic 2+ and posterior subcapsular 2+ in the right eye, nuclear sclerotic 1+ in the left eye). Dilated fundus examination of the right eye showed an epiretinal membrane, which was confirmed on optical coherence tomography, and left eye fundus was unremarkable.

**Fig. 1:**
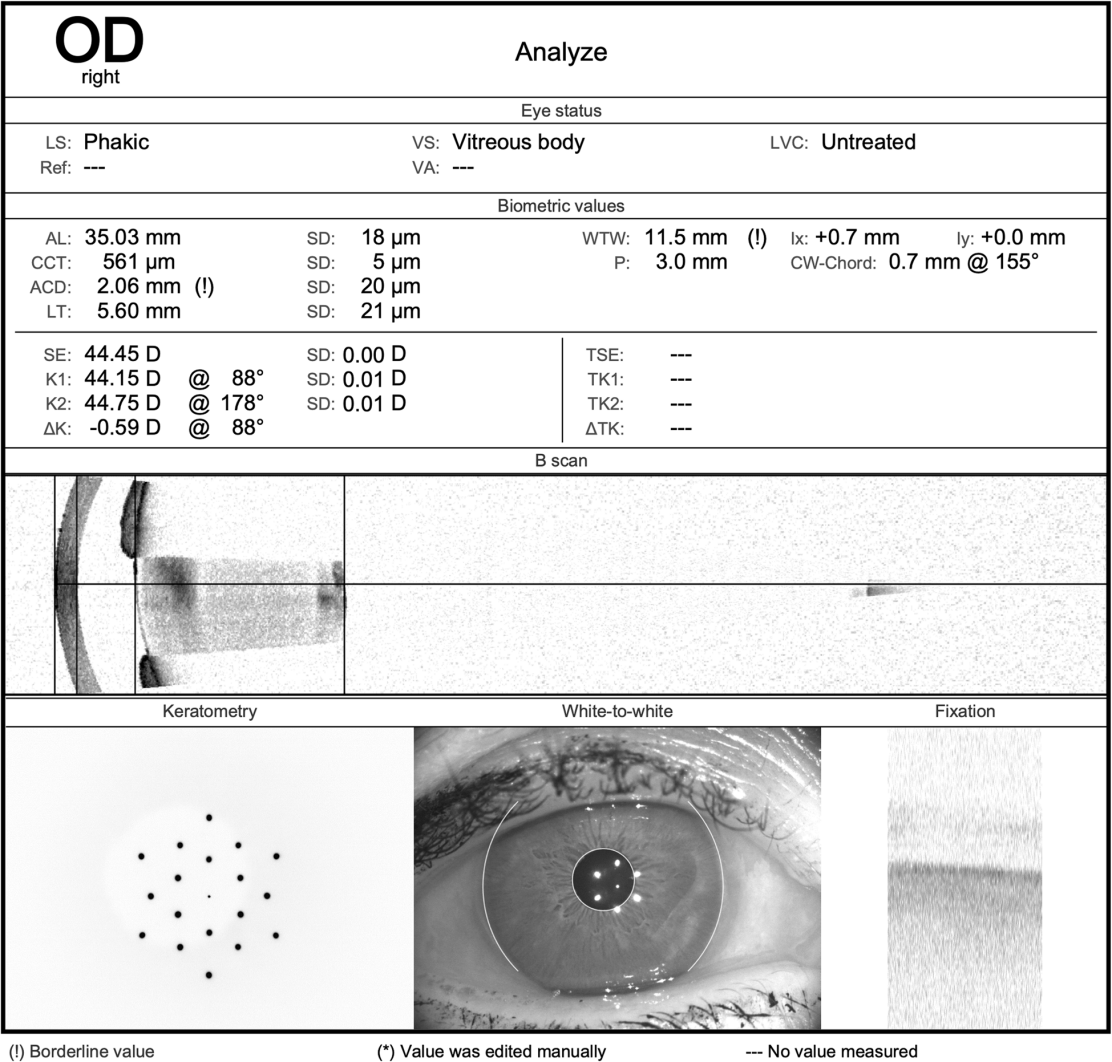
Pre-operative SS-OCT biometry of the right eye

**Fig. 2:**
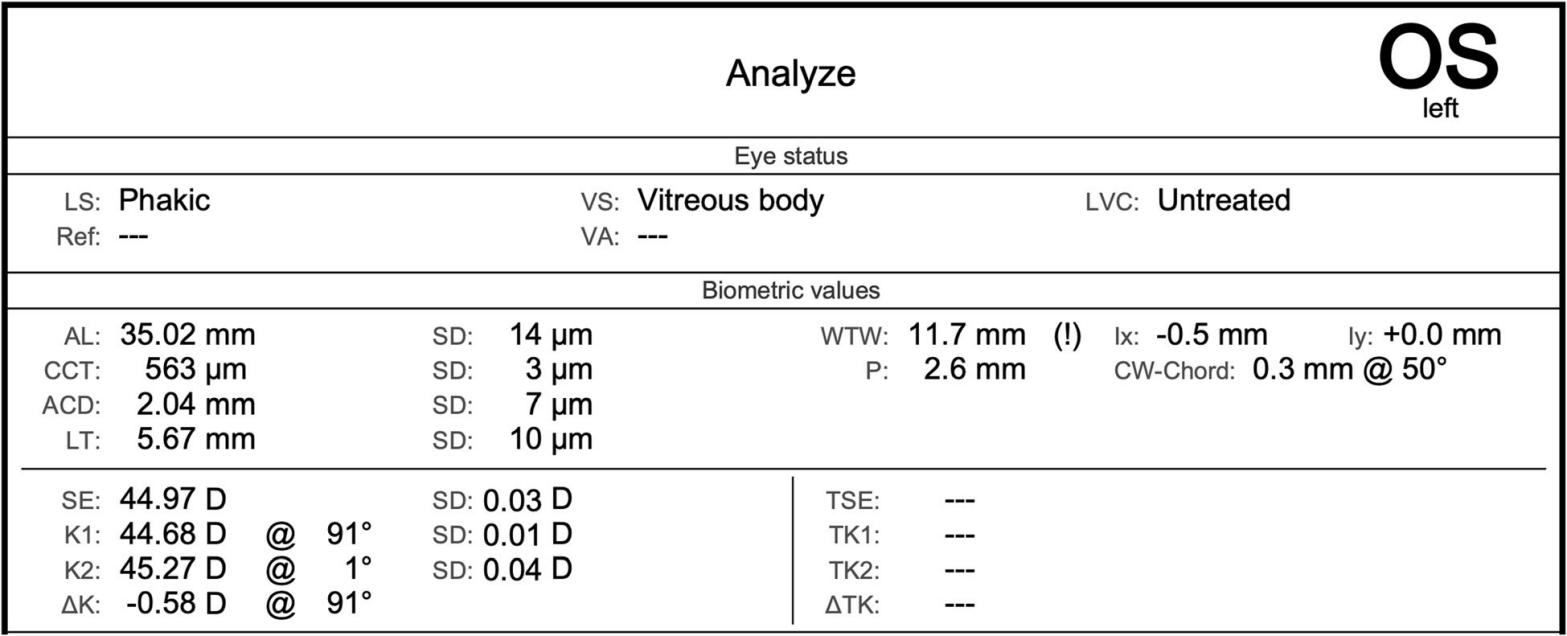
Pre-operative SS-OCT biometry of the left eye

During nurse-led pre-assessment clinic, SS-OCT biometry (IOLMaster 700, Carl Zeiss Meditec) was performed, which measured the axial lengths as 35.03 mm in the right eye and 35.02 mm in the left eye [Figs. 1 and 2]. Due to the mismatch between the clinical parameters and biometry, the measurements were repeated three times that day, confirming the initial measurements. All axial length readings were within the manufacturer’s acceptable standard deviation of repeatability, suggesting quality capture.

Utilising the Haigis formula, the Alcon MA60MA − 5.00 dioptre intraocular lens was requested in preparation for surgery in the right eye, aiming for − 0.55D refraction post-operatively. During the consent, the risk of refractive surprise was emphasised, considering her long axial length. Phacoemulsification and intraocular lens implantation was performed under local anaesthesia ten days following the pre-assessment and was uncomplicated.

Post-operatively, the patient complained of substantially worse vision in the operated eye. Corrected distance visual acuity was counting fingers. Anterior segment examination was unremarkable – the lens was well-centred in an intact, non-distended capsular bag, with no retained ophthalmic viscosurgical device. Intraocular pressure, measured by applanation tonometry, was 13 mmHg. Dilated fundus examination demonstrated a superotemporal branch retinal vein occlusion with associated macular oedema. Autorefraction was + 14.37 DS / + 2.25 DC × 142.

**Fig. 3:**
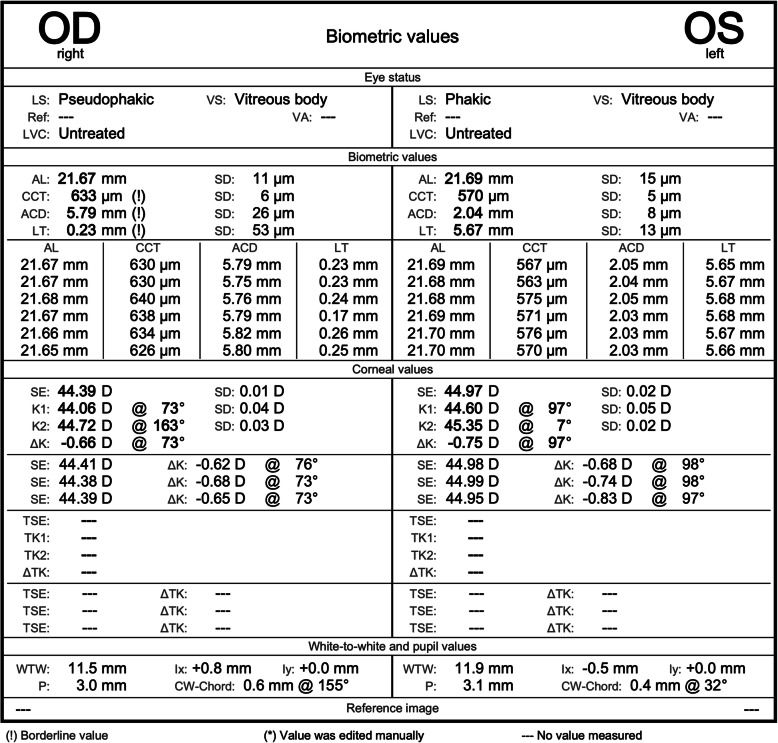
Post-operative SS-OCT biometry of both eyes

Biometry was repeated with the same biometer [Fig. 3] and demonstrated different axial lengths in both eyes: 21.67 mm in the right eye and 21.69 mm in the left eye. The patient subsequently underwent an intraocular lens exchange. The original lens was removed and a Bausch&Lomb EyeCee + 27.5 dioptre intraocular lens (aiming for + 0.17D refraction post-op, utilising the Sanders/Retzlaff/Kraff/T formula) was inserted into the intact bag. The following fortnight, corrected distance visual acuity was 20/320 in the right eye. Autorefraction was 0.00 DS − 0.45 DC × 46. The fundus was unchanged and she was listed for monthly intravitreal aflibercept injections to the right eye for management of macular oedema, secondary to the branch retinal vein occlusion.

On subsequent investigation, it was then established that the biometer technician from the manufacturer had serviced the machine the day following the initial biometry, at the request of nursing staff operating the biometer. They had noted variability in readings with other patients – initially exaggerated axial lengths which were then measured as within the normal range on repeat testing that day. On analysis of the biometer, the only abnormality detected was a ‘smear’ on its optical surface, which they had removed with a fine brush.

## Discussion and conclusions

There is extensive literature reporting accurate and consistent optical biometry providing excellent visual and refractive outcomes. SS-OCT offers the additional benefit of acquiring measurements even in dense cataracts [9]. As far as we are aware, this is the first case report of optical biometry measuring the axial length as being abnormally long in both eyes, despite repeated measurements and good agreement between eyes. Unfortunately, the error was not discovered until after the initial cataract operation, by which point the patient not only had a significant refractive surprise but had also suffered the misfortunate of a branch retinal vein occlusion, with associated macular oedema.

During the pre-operative assessment, the clinical and refractive picture was not consistent with the axial length measured, hence the request to repeat the initial biometry was reasonable. Currently, the National Institute for Health and Care Excellence (United Kingdom) guidelines do not mandate repeating biometry [10], but the Royal College of Ophthalmologists (United Kingdom) guidelines do for axial lengths >26mm [11]. Despite the repeat measurements, the axial length was still measured as reliable and abnormally long.

As is expected with wrong intraocular lens insertion, a root cause analysis occurred with the aim of preventing this from occurring again [12]. During the analysis, we struggled to account for the significant difference in the measured axial lengths pre- and post-operatively. It was unlikely to be a patient factor (such as a media opacity overlying the cornea, or retinal changes) in view of both eyes being equally affected and the biometry results being repeatedly consistent and reliable. Lastly, we came to biometer error – either the software, hardware or both. The manufacturer of the device confirmed that the residue discovered on the optical service of our biometer was responsible. Interestingly, anterior chamber depth remained constant in all measurements, apart from in the post-operative pseudophakic right eye, as would be expected due to the removal of the cataract.

To substantiate this theory, our root cause analysis identified that multiple patients who had biometry performed the same day as our patient, using the same biometer, had inflated axial lengths (> 30 mm). However, unlike the patient in this case report, repeat axial length measurements was highly variable. The discrepancies in these readings resulted in a biometer technician review the following day, the error being identified and accurate biometry performed prior to their cataract surgery.

In summary, this is a unique case of SS-OCT biometry consistently overestimating the axial length, due to residue on the optical surface of the biometer, resulting in the incorrect intraocular lens insertion. Fortunately, the correct lens was exchanged successfully, optimising the refractive outcome. The manufacturer of our biometer recommends regular maintenance of the optical surface. However, there is no clear guidance as to the optimal frequency, as there is the theoretical risk of damage if it is performed too frequently. We advocate cleaning the optical surface at the beginning of every pre-assessment clinic, and also highlighting users to our case, to minimise refractive surprise. If there is uncertainty with the axial length not matching clinical parameters, despite having reliable scans, then consider performing biometry on another day and/or using a different modality to confirm the measurement.

## Data Availability

Not applicable.

## Abbreviations

SS-OCT: Swept-source optical coherence tomography
